# Impact of metabolic indices of ^18^F-fluorodeoxyglucose positron emission tomography/computed tomography on post transplantation recurrence of hepatocellular carcinoma

**DOI:** 10.1007/s00432-022-04009-x

**Published:** 2022-04-22

**Authors:** Astrid Bauschke, Annelore Altendorf-Hofmann, Lukas Brückner, Robert Drescher, Martin Freesmeyer, Utz Settmacher

**Affiliations:** 1 Department of General, Visceral and Vascular Surgery, University Hospital Jena, Am Klinikum 1, Erlanger Allee 101, 07740 Jena, Germany; 2 Department of Nuclear Medizine, University Hospital Jena, Am Klinikum1, 07740 Jena, Germany

**Keywords:** Liver transplantation, HCC, Long-term survival, ^18^F-FDG PET/CT

## Abstract

**Background:**

Tumor recurrence is the leading cause of death after liver transplantation in patients with hepatocellular carcinoma. There is an ongoing debate as to whether metabolic indices such as tumor to liver standardized uptake value ratio in ^18^F-fluorodeoxyglucose positron emission tomography/computed tomography of the primary tumor can identify patients outside the Milan criteria with as low recurrence rates as patients inside Milan and thus should be added to the established prognostic factors.

**Methods:**

This retrospective study analyzes 103 consecutive patients who underwent ^18^F-fluorodeoxyglucose positron emission tomography/computed tomography before liver transplantation for hepatocellular carcinoma using data of clinical tumor registry. Primary endpoints were overall survival and 10-year cumulative recurrence rates.

**Results:**

Tumor to liver standardized uptake value ratio of the primary tumor was statistically significant higher in Milan out tumors, “up-to-seven” out tumors, grade 3 tumors, α- fetoprotein level >400 ng/ml and lesions > 5cm in diameter. Factors with statistically significant influence on the 10- year overall survival in the univariate analysis were Milan, up-to-seven” criteria, number of lesions and pT-category. COX regression analysis did not show independently statistically significant factors for 10-year overall survival. Milan, “up-to-seven” criteria, grade, pV, number of lesions, size of lesion, pT-category, tumor to liver standardized uptake value ratio influenced 10-year cumulative recurrence rates statistically significant. Tumor to liver standardized uptake value ratio, grade and pT-category proved to be independently statistically significant factors for 10-year cumulative recurrence rates.

**Conclusions:**

Our study suggests that tumor to liver standardized uptake value standardized uptake value ratio in ^18^F-fluorodeoxyglucose positron emission tomography/computed tomography is an independent prognostic factor in transplanted patients with hepatocellular carcinoma. If we focus on preoperative findings, such as tumor size, tumor number and AFP value adding the information given by TLR of ^18^F-FDG PET/CT allows to estimate the risk of tumor recurrence more accurate than the established classifications Milan and UTS. Therefore, it may add valuable information to other preoperative findings, such as tumor size, tumor number and AFP level.

## Introduction

Tumor recurrence is the leading cause of death after liver transplantation (LT) in patients with hepatocellular carcinoma (HCC) in cirrhosis. In 1996, Milan criteria were introduced by Mazzaferro et al. (1996) and this classification is still recommended by guidelines for assigning patients exceptional Meld points or for initial listing for liver transplantation. It is well known that a certain patient population “outside” Milan can be found that show similar low recurrence rates as patients “inside” Milan. There is an ongoing debate as to whether biological markers, such as alpha-fetoprotein (AFP), Des-gammo-carboxy prothrombin (DCP), grading, neutrophil–lymphocyte ratio (Halazun et al. 2009) or downstaging after initial presentation with disease outside the Milan criteria (Bauschke et al. 2020; Gordon-Weeks et al. 2011; Millonig et al. 2007; Otto et al. 2006; Pavel and Fuster 2018; Ravaioli et al. 2008; Roayaie et al. 2004) should be considered to refine criteria for transplantation.

The diagnostic potential of ^18.^F-fluorodeoxyglucose positron emission tomography/computed tomography (^18^F-FDG PET/CT) in the evaluation of transplant candidates is well established for extrahepatic tumor. However, the sensitivity of ^18^F-FDG PET/CT for HCC is lower than in metastatic liver cancer or cholangiocellular carcinoma (CCC) (Iwata et al. 2000). In recent years, the use of volumetric indices in PET/CT has been more frequent because they may reflect location of cancerous tissue as well as metabolic activity. This combination of metabolic activity and computed tomography (CT) images is supposed to discriminate more precisely between physiologic and malignant FDG uptake and may support physicians in the calculation of recurrence risk more accurately.

The study analyzes the value of 18F-FDG PET/CT for the identification of patients with HCC in cirrhosis and the tumor biology after LT.

## Materials and methods

This study in human subjects was conducted with consent of the local ethics committee (reg.-no.:2020-1827-Daten) in accordance with national law and the Declaration of Helsinki of 1975 (in the current form).

### Patients

Here we analyze 103 consecutive patients who underwent ^18^F-FDG PET/CT in our hospital before liver transplantation for HCC from 2009 to 2019. Patient data, HCCs, treatment and follow-up were extracted from standard medical records. Data not found in the standard medical records were completed by contacting clinicians.

Diagnostic procedures were applied following current European guidelines for the diagnosis and treatment of hepatocellular carcinoma (Llovet et al. 2012). Decisions about diagnosis and treatment were made by the tumor board with participation of hepatobiliary surgeons, radiologists, oncologists, nuclear medicine physicians and radiotherapists. The results of ^18^F-FDG PET/CT scans were not used for patient selection at any time during the study period and the policy in this context has not been changed throughout the study period.

We analyzed the morphological data of the tumor load in pre-transplant computed tomography scans (CT) or magnetic resonance imaging (MRI) scans, α-fetoprotein (AFP) (ng/ml) level, TNM stage (Brierley et al. 2017), stage of underlying liver disease (Child–Pugh-stage) and use of loco-regional therapy before liver transplantation. The categorization of patients inside/outside the Milan criteria, inside/outside UTS, and AFP-level, are given before bridging therapy and before liver transplantation.

In cases of sufficient liver function bridging procedures, such as liver resection, local ablative procedures (transarterial chemoembolization (TACE), radio frequency ablation (RFA), Yttrium90 radio embolization (Y^90^RE), tomotherapy, in combination with systemic therapy with thyrosinkinase inhibitor were employed. All these interventions were continued for as long as residual tumor was identified and monitored radiologically in 90 days intervals. In cases of residual vital tumor, the procedures were repeated and combined.

### Calculation of standardized uptake value (SUV) max, standardized uptake value (SUV) mean and tumor to liver ratio (TLR)

Whole-body ^18^F-FDG PET/CT scan was performed before LT as described recently (Winkens et al. 2021). The maximum SUV (SUVmax) of a hepatic tumor was measured by drawing a volume-of- interest (VOI) over the target lesion with reference to PET, contrast- enhanced CT, and/or MRI images. In case of multiple lesions, the highest SUVmax was used as a representative value. Tumor to liver SUV ratio (TLR) was calculated as the ratio of SUVmax of the tumor to SUVmean of normal liver tissue. A receiver operating characteristic (ROC) analysis was performed to define the optimal F-18-FDG uptake value cut-off to predict tumor recurrence.

### Statistical methods

All statistical analyses were performed using SPSS 26.0 software (IBM, Chicago, IL, USA) software. Distributions of variables were evaluated using the Chi-square test, Fisher’s exact test or Mann–Whitney *U* test, as indicated. Cumulative recurrence rates were calculated from the date of liver transplantation to first clinical diagnosis of tumor recurrence. Patient deaths unrelated to HCC recurrence were censored. Cumulative recurrence curves were created using the Kaplan–Meier method. Median follow-up time was calculated using the reverse Kaplan–Meier method. Differences in recurrence rates as well as significant and independent predictors of recurrence were identified by Cox proportional hazard analysis. Statistical significance was defined as a *p* value < 0.05 for all analyses.

## Results

From 2009 to 2019, 103 patients underwent ^18−^F-FDG-PET/CT in our hospital before liver transplantation for HCC. Patients’ age at transplantation was median 62 years (23–71 years). Morphological tumor load was inside Milan in 54 (52%) patients and outside Milan in 49 (48%) patients. 79 (77%) patients received a liver from deceased donors. 24 patients got a split from a living donor (all of them were right lobes). The waiting time was median 9 months (0–45 months) for LT from deceased donor and 6 months (0–34 months) for the living donations. The median interval between PET/CT scan and liver transplantation was 6 months (0–41 months). Further details on patients, tumor load and treatment are shown in Table 1.

**Table 1:** Patient under study

Item	Strata	*n*	%	Tumor to liver SUV ratio				
				Quartiles			Maximum	*p*
				25	50	75		
Age	< 60 years	38	37	1.00	1.00	1.78	3.32	0.117
	≥ 60 years	65	63	1.00	1.00	1.98	8.74	
Sex	Male	91	88	1.00	1.00	1.85	8.74	0.868
	Female	12	12	1.00	1.00	2.00	4.23	
Milan	In	58	56	1.00	1.00	1.61	8.74	0.033
	Out	45	44	1.00	1.42	2.08	6.83	
UTS	In	57	57	1.00	1.00	1.56	8.74	0.015
	Out	46	46	1.00	1.45	2.10	6.83	
Bridging before Transplantation	No	36	35	1.00	1.00	2.11	8.74	0.943
	Yes	67	65	1.00	1.00	1.85	6.83	
AFP^a^	0–399 ng/ml	87	90	1.00	1.00	1.79	4.23	0.001
	≥ 400 ng/ml	10	10	1.68	2.32	3.99	8.74	
Grade	Grade 1–2	88	85	1.00	1.00	1.79	8.74	0.023
	Grade 3	15	15	1.00	2.08	3.04	6.83	
Type of transplantation	Diseased donor	79	77	1.00	1.00	1.85	8.74	0.187
	Living donor	24	23	1.00	1.57	2.09	3.32	
Microvascular invasion (pV)	pV0	82	80	1.00	1.00	1.92	8.74	0.424
	pV1	21	20	1.00	1.00	1.79	3.32	
Number of lesions	solitary	51	50	1.00	1.00	1.68	8.74	0.108
	multipel	52	50	1.00	1.00	2.08	6.83	
Size of lesion	< 5 cm	72	72	1.00	1.00	1.66	4.23	0.007
	≥ 5 cm	31	31	1.00	1.71	2.14	8.74	
Child stage	Child A/B	92	89	1.00	1.00	1.89	8.74	0.694
	Child C	11	11	1.00	1.00	2.09	4.20	
pT-category	pT1/2	87	85	1.00	1.00	1.81	8.74	0.064
	pT3/pT4	16	15	1.00	1.68	2.76	4.20	
Total		103	100	1.00	1.00	1.91	8.74	

^a^6 missing

Of all 103 patients, 25 had a bridging procedure before PET/CT, another 42 patients had at least one bridging procedure after ^18^F-FDG PET/CT and before liver transplantation. Three of the 25 patients who were bridged before ^18^F-FDG PET/CT had a complete pathological response (no vital tumor in the explanted liver), but none of these three patients had a complete radiological response before ^18^F-FDG PET/CT.

Median follow-up time after LT was 79 months (0–139 months). During the interval 48 patients died, 9 of them in the postoperative interval, 18 due to HCC recurrence. Three patients died from malignant second tumor (lung cancer in 1, ENT area in 2), and 18 died from tumor unrelated causes.

SUV_max_ in tumor tissue ranged from 1.1 to 23.6 with a median of 2.6. SUV_mean_ in non-tumor liver tissue ranged from 1.1 to 3.6 with a median of 2.3. Median tumor to liver SUV ratio was 1.0 (1.0–8.74).

In Table 1, Quartiles of Tumor to liver SUV ratio are given for all listed subgroups. The Tumor to liver SUV ratio values were compared by Mann–Whitney *U* test. Tumor to liver SUV ratio (TLR) of the primary tumor was statistically significant higher in Milan out tumors (*p* = 0.018), “up-to-seven” out tumors (*p* = 0.015), grade 3 (*p* = 0.023), patients with AFP level > 400 ng/ml (*p* < 0.001) and lesions of a diameter of 5 cm and more (*p* = 0.007).

All other factors (age, sex, bridging therapy before transplantation, type of transplantation, microvascular invasion, number of tumors, Child–Pugh-stage, pT-category, necrosis in the tumor) did not show a statistically significant dependence on the Tumor to liver SUV ratio.

A ROC analysis was performed to define the optimal cut-off for the Tumor to liver SUV ratio to predict tumor recurrence. In the present study, we chose a cut-off value of > 1.38, giving a sensitivity of 70.0% and a specificity of 67.6%. A cut-off value of 1.80 or 2.00 gives sensitivity of 57% and 48% and specificity of 79% and 83%, respectively.

### Analysis of overall survival

Patients who died in the first 3 months were excluded from survival and recurrence analysis resulting in 94 patients for long-term analysis. All 94 patients were followed up until death or until 31st December 2020. To date, 5 patients lived for more than 10 years after transplantation, 34 for more than 5 years. All living patients have been followed up for at least 1 year. Five patients died from HCC recurrence during the first year after LTX.

Median survival time after transplantation was 106 months, the overall 5- and 10 year-survival rates were 66% and 34%, respectively.

Univariate analysis found only four factors with statistically significant influence on 10 year overall survival: Milan (*p* = 0.018), “up-to-seven” (*p* = 0.044), number of lesions (*p* = 0.011) and pT-category (*p* = 0.047). Milan, number of lesions and pT-category were included in a multivariate COX regression analysis, which did not show independent statistically significant factors for 10 year overall survival.

A second multivariate COX analysis including the preoperative accessible Milan, AFP level and TLR showed only Milan to be an independent statistically significant factors for 10 year overall survival (*p* = 0.044, Exp(B) 2.127 (1.020–4.436)).

### Analysis of cumulative recurrence rate

The majority of the 23 recurrences (70%) occurred in the first two years after transplantation, but there was also a substantial number of later recurrences. The median interval to tumor relapse was 15 months (2–84 months).

Recurrence was intrahepatic in 6 patients and extrahepatic in 17 patients. Sites of extrahepatic recurrence were lung (6 patients), bones (5 cases), adrenal gland (2 patients), peritoneum (2 patients), abdominal wall (1 patient) and lymph nodes (1 patient). Tumor recurrence was treated with curative intent in 8 patients. Pulmonary metastases were resected in 3 patients, adrenal metastases in 2 patients, and metastases in lymph nodes and metastases in the abdominal wall and local recurrence in the liver in one patient each.

5- and 10 year cumulative recurrence rates were 28% and 34%.

**Fig. 1:**
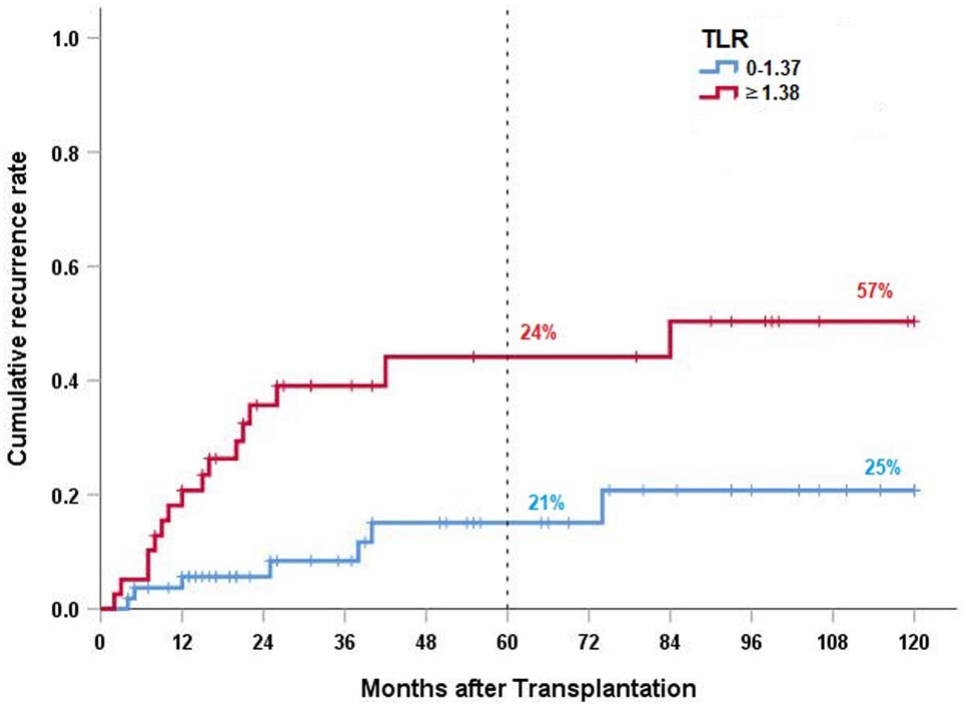
Cumulative recurrence rate according to tumor to liver SUV ratio

Age, sex, bridging before PET/CT and Child stage did not influence cumulative 10-year recurrence rates statistically significant but Milan, “up-to-seven” grade, microvascular invasion, AFP-level, number of lesions, size of lesion, pT-category, Tumor to liver SUV ratio did (Fig. 1). Details for all recurrences are shown in Table 2a.

**Table 2:** **a** Univariate and multivariate analysis of cumulative recurrence rates for all recurrences. **b** Univariate and multivariate analysis of cumulative rates of early recurrence

All patients, *n* = 94		Univariate		Cut off	Multivariate Model 1		Multivariate Model 2	
Prognostic factor	Strata	*p*	Exp(B) (95% CI)		*p*	Exp(B) (95% CI)	*p*	Exp(B) (95% CI)
Age	< 60 years/ ≥ 60 years	0.975	1.013 (0.437–2.350)					
Sex	female/male	0.394	0.532 (0.124–2.272)					
Milan	In/out	**0.001**	4.344 (1.780–10.602)					
UTS	In/out	**0.001**	5.056 (1.984–12.887)					
Score	In/out	**0.002**	6.622 (1.962- 22.354)					
Bridging before transplantation	Yes/no	0.609	0.784 (0.309–1.990)					
AFP	0–399 / ≥ 400 ng/ml	**0.008**	3.939 (1.426–10.878)	1.38 1.80 2.00			**0.036** 0.085 0.078	3.165 (1.080–9.276) 2.643 (0.873–7.999) 2.831 (0.891–8.996)
Grade	Grade 1–2/Grade 3	**0.016**	3.179 (1.242–8.131)	1.38 1.80 2.00	**0.044** 0.055 0.066	2.780 (1.029–7.513) 2.701 (0.980–7.448) 2.690 (0.938–7.717)		
Microvascular invasion	pV0/pV1	**0.008**	3.099 (1.340–7.166)					
Number of lesions	Solitary/multiple	**0.014**	3.066 (1.254–7.496)					
Size of lesion	< 5 cm/ ≥ 5 cm	**0.014**	2.799 (1.232–6.362)					
Child stage	Child A-B/Child C	0.653	1.321 (0.392–4.450)					
pT-category	pT0-2/pT3-4	** < 0.001**	5.326 (2.270–12.496)	1.38 1.80 2.00	** < 0.001** ** < 0.001** ** < 0.001**	5.564 (2.272–13.622) 5.948 (2.437–14.518) 5.580 (2.278–13.667)	**0.001** **0.002** **0.002**	5.192 (1.951–13.818) 4.788 (1.791–12.795) 4.728 (1.746–12.800)
Tumor to liver SUV ratio	< 1,38/ ≥ 1,38 < 1,80/ ≥ 1,80 < 2.00/ ≥ 2.00	**0.005** **0.004** **0.004**	3.562 (1.463–8.672) 3.322 (1.454–7.590) 3.392 (1.491–7.716)	1.38 1.80 2.00	**0.031** **0.033** 0.085	2.783 (1.096–7.067) 2.606 (1.081–6.280) 2.225 (0.895–5.533)	0.056 **0.024** 0.095	2.667 (0.975–7.297) 3.145 (1.160–8.529) 2.366 (0.862–6.499)

All patients, *n* = 94		Univariate		Multivariate Model 1			Multivariate Model 2	
Prognostic factor	Strata	*p*	Exp(B) (95% CI)	Cut off	*p*	Exp(B) (95% CI)		
Age	< 60 years/ ≥ 60 years	0.545	0.737 (0.274–1.981)					
Sex	female/male	0.398	0.417 (0.055–3.164)					
Milan	In/out	**0.002**	7.493 (2.132–26.334)					
UTS	In/out	**0.001**	11.201 (2.540–49.388)					
Score	In/out	**0.048**	58.229 (1.029–3296.435)					
Bridging before transplantation	Yes/no	0.574	0.723 (0.233–2.241)					
AFP	0–399 / ≥ 400 ng/ml	**0.013**	4.282 (1.360–13.478)	1.38 1.80 2.00			**0.032** 0,066 0,091	3.883 (1.123–13.426) 2,922 (0,933–9,152) 2,760 (0,849–8,975)
Grade	Grade 1–2/Grade 3	**0.013**	3.874 (1.336–11.235)	1.38 1.80 2.00	0.234 0,087 0,097	1.970 (0.645–6.021) 2,930 (0,856–10,025) 2,910 (0,824–10,283)		
Microvascular invasion	pV0/pV1	**0.009**	3.747 (1.394–10.072)					
Number of lesions	Solitary/multiple	**0.011**	5.141 (1.464–18.055)					
Size of lesion	< 5 cm/ ≥ 5 cm	**0.028**	2.999 (1.124–8.003)					
Child stage	Child A-B/Child C	0.714	1.319 (0.300–5.806)					
pT-category	pT0-2/pT3-4	**0.001**	5.183 (1.926–13.945)	1.38 1.80 2.00	**0.009** **0,002** **0,003**	3.829 (1.400–10.471) 5,458 (1,883–15,819) 5,258 (1,758–15,725)	**0.001** **0,002** **0,005**	6.147 (2.116–17.859) 4,915 (1,770–13,653) 4,525 (1,591–12,872)
Tumor to liver SUV ratio	< 1,38/ ≥ 1,38 < 1,80/ ≥ 1,80 < 2.00/ ≥ 2.00	**0.004** **0.002** **0.001**	6.488 (1.848–22.775) 5.330 (1.852–15.345) 5.597 (2.033–15.410)	1.38 1.80 2.00	**0.023** 0.087 0.057	4.555 (1.234–16.817) 3.821 (1.122–13.009) 3.217 (0.968–10.689)	**0.043** **0.023** **0.034**	3.865 (1.041–14.346) 3.610 (1.193–10.926) 3.293 (1.093–9.921)

Bold values indicate *p* < 0.05

The results for univariate and multivariate COX analyses depended on the three different cut-off values are presented in Table 2a, b.

16 of the 23 recurrences were early recurrences, that means, they occurred in the first 2 years after transplantation. We repeated the analyses given in Table 2a for early recurrences. In univariate as well as in multivariate analyses we saw only marginal differences in the results (Table 2b).

To achieve reliable results in multivariate COX analyses for the 23 patients with recurrence, a maximum of three factors should be used (Peduzzi et al. 1995). pT-category (because this factor takes number and size of lesions and vascular invasion into account), tumor to liver SUV ratio and grade were chosen. All three factors proved to be independent statistically significant factors for 10 year cumulative recurrence rates (Table 2a). For a second multivariate COX analysis, we chose pT-category, tumor to liver SUV ratio and pre-transplant AFP-level. In this analysis, pT-category and pre-transplant AFP-level were independent statistically significant factors for 10 year cumulative recurrence rates but tumor to liver SUV ratio was not (Table 2a).

A multivariate COX analysis only including the preoperative accessible Milan, AFP level and TLR showed only Milan to be an independent statistically significant factors for 10 year cumulative recurrence rates (*p* = 0.043, Exp(B) 2.838 (1.035–7.781)).

After stratification for Milan criteria, we repeated the univariate Cox Analysis for cumulative recurrence rates depending on the TLR. For a limited numbers of patients, we saw no statistically significant differences (*p* = 0.069, 4.047 (0.898–18.236) and *p* = 0.234, 1.999 (0.640–6.247), respectively) between patients Milan in and Milan out.

**Fig. 2:**
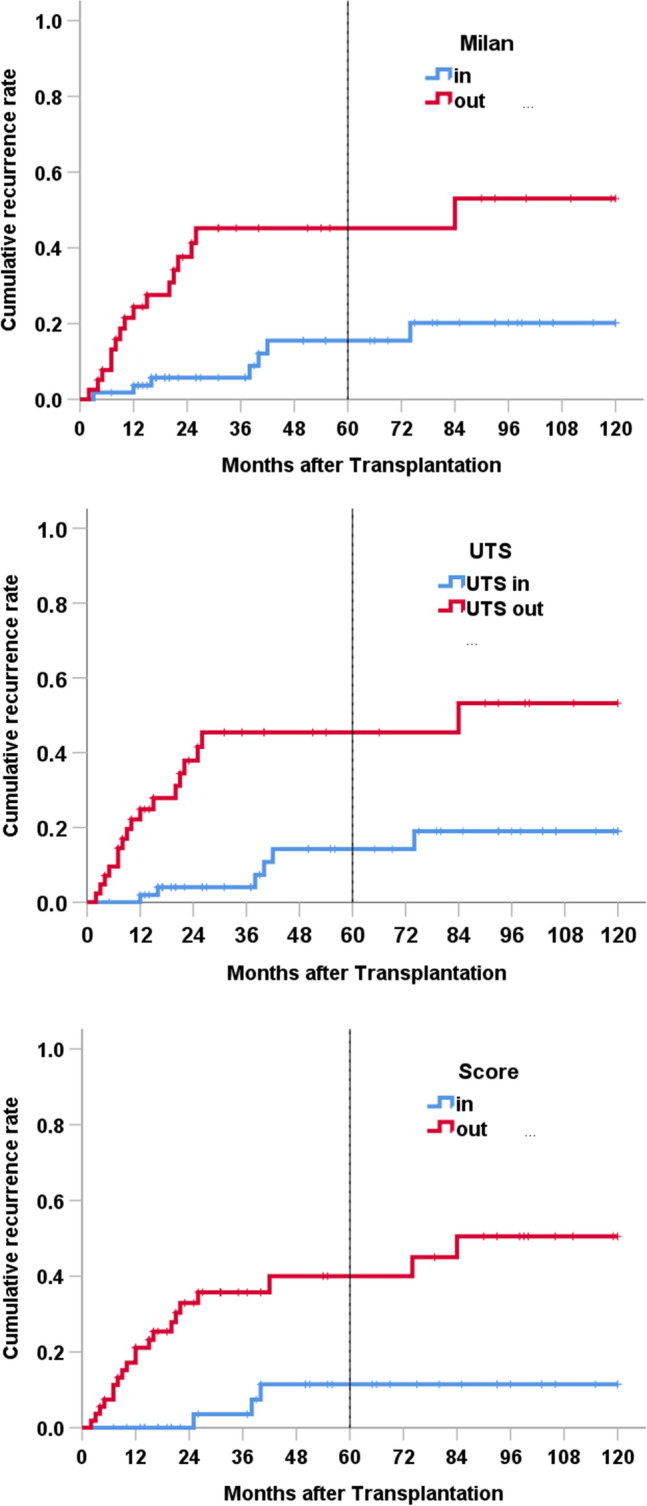
Cumulative recurrence rates according to Milan, UTS and risk score

A score including the preoperative accessible values Diameter, number of lesions and AFP level was calculated. 40 cases with TLR < 1.38, Diameter < 5 cm, 1 to 6 lesion and AFP < 400 ng/ml were defined as to be “low risk”, 54 others “high risk”. All early recurrences and only three patients with recurrences at 25 months, 38 months and 40 months were classified into the low risk group. Therefore, the classification for risk of recurrence was slightly better than the grouping given by Milan or UTS (Fig. 2).

## Discussion

In our study, tumor to liver SUV ratio (TLR) of the primary tumor was statistically significantly higher in Milan out tumors, “up-to-seven” out tumors, Grade 3 tumors, AFP level > 400 ng/ml and lesions of a diameter of 5 cm or more.

Like us, many investigators found TLR to be statistically significantly higher in tumors with negative prognostic factors, such as high grade and microvascular invasion (Bailly et al. 2016), high preoperative AFP level, Milan out, University of California, San Francisco (UCSF) out, large tumor size, major vessel invasion, and serosal invasion (Lee et al. 2013; Ye et al. 2017). Therefore, they presumed ^18^F-FDG PET/CT could be a noninvasive diagnostic tool to identify HCCs with negative prognostic factors and a high incidence of tumor recurrence.

Like others, we found an independently statistically significant influence of metabolic activity in ^18^F-FDG PET/CT on cumulative recurrence rate. Therefore, it can add valuable information to other preoperative findings, such as tumor size, tumor number and AFP value.

Seo et al. were among the first authors who reported a prognostic usefulness of ^18^F-FDG PET/CT in transplanted patients with HCC (Seo et al. 2007). They found that in HCC patients with an uptake of ^18^F-FDG in a primary HCC lesion equal to the uptake in a normal liver the 2 year recurrence-free survival rate was significantly higher than that of PET patients with an increased uptake of ^18^F-FDG in the primary HCC lesion.

Since then, many studies reporting the influence of metabolic activity on overall survival or recurrence rates were undertaken. They either used a semi-quantitative classification (Kornberg et al. 2017; Takada et al. 2017) or the TLR (Detry et al. 2015; Lee et al. 2013; Ye et al. 2017).

Integrated PET/CT, combining a full-ring-detector clinical PET scanner with a multi-detector-row helical CT scanner has made it possible to acquire both metabolic and morphologic imaging data with a single device in one diagnostic session, and has been demonstrated to show precise anatomic location of suspicious areas of increased FDG uptake.

In our study, factors which had a statistically significant influence on 10 year overall survival in univariate analyses were Milan, up-to-seven”, number of lesions and pT-category. Multivariate COX regression analysis did not show independently statistically significant factors for 10 year overall survival.

In Table 3 key data from studies about the prognostic value of ^18^F-FDG PET/CT are compared. Noticeably, some authors report short follow-up intervals (Yang et al. 2006), which might miss a considerable proportion of recurrences. Another surprising point is that not all studies analyzed the influence of Milan criteria on the cumulative recurrence rate (Kang et al. 2019; Yang et al. 2006) and others did not find a statistically significant influence of Milan on recurrence rates (Kim et al. 2016; Lee et al. 2013; Ye et al. 2017) in the univariate analysis.

**Table 3:** Studies with univariate COX analyses of recurrence rates

	Present study	Yang et al. (2006)**	Lee et al. (2013)*^,c^	Detry et al. (2015)	Kim et al. (2016)	Hsu et al. (2016)	Ye et al. (2017)^d^	Kang et al. (2019)^a^
Period under study	2009–2019	2000–2004	2005–2011	2006–2011	2008–2012	2006–2014	2006–2013	2005–2013
Patients under study	94	38	191	27	110	147	103	239
Patients with “positive” PET	39 (42%)	13 (34%)	55 (29%)	8 (30%)	39 (35%)	30 (20%)	78 (76%)	–
Median follow up (months)	79 (3–122)	19 (5–40)	28 (1–79)	26^b^	46^b^	26	26^b^	53 (5–131)
Patients with recurrence	23 (25%)	11 (29%)	38 (20%)	5 (19%)	30 (27%)	18 (12%)	53 (52%)	74 (31%)
Cutoff value of SUV ratio	1.38	1	1	1.15	1.16	2	1	2.8
Milan	*p* = 0.001	–	*p* < 0,001	*p* = 0.21	*p* = 0.004	*p* = 0.830	*p* < 0.001	–
UTS	*p* = 0.001	–	–	–	–	–	–	–
Size of lesions	*p* = 0.013	–	*p* < 0,001	*p* = 0.05	*p* < 0.001	*p* = 0.347	–	*p* < 0,001
Number of lesions	*p* = 0.013	–	–	*p* = 0.99	*p* = 0.012	*p* = 0.795	*p* = 0.005	*p* < 0,001
pT-category	*p* < 0.001	–	–	–	–	*p* = 0.032	–	–
AFP	*p* = 0.008	–	*p* = 0,001	*p* = 0.47	–	*p* = 0.894	*p* = 0.001	*p* < 0,001
Tumor to liver SUV ratio	*p* = 0.005	*p* = 0.003	*p* < 0,001	*p* = 0.01	*p* < 0.001	*p* < 0.001	*p* = 0.011	*p* < 0,001

Not investigated *3-year rates presented **2-year rates presented

^a^Multicentric

^b^Mean follow-up

^c^Only living donor liver transplantation

^**d**^Only patients with HBV-related HCC

Recurrence rates vary between 12 (Hsu et al. 2016) and 52% (Ye et al. 2017). The number of patients with recurrence limits the informative value of multivariable analyses, because results of studies having fewer than ten events per variable analyzed should be interpreted with caution (Peduzzi et al. 1995).

In our study, 5-year and 10 year cumulative recurrence rates are 27% and 34%, respectively. A univariate analysis found that they were statistically significantly influenced by Milan, grade, pV, number of lesions, size of lesions, pT-category, and tumor to liver SUV ratio (Table 3).

After the introduction of the Milan criteria, multiple other classifications were proposed. They are predominantly based on the morphologic tumor burden, measured by number and diameter of the lesions, sometimes complemented by variables of liver function or preoperative AFP value (Bauschke et al. 2017).

Two study groups from South Korea proposed scores including the findings in PET/CT in new scores for estimation of the prognosis after living donor liver transplantation for HCC. Both yield results comparable to the Milan criteria (Kang et al. 2019; Lee et al. 2016).

Table 4 lists studies with PET/CT using multivariate Cox regression analysis to identify independently statistically significant factors for cumulative recurrence rates. Only in two cases, the number of events per variable analyzed exceeds 5.

**Table 4:** Studies with multivariate COX analyses of recurrence rates

	Present study	Lee et al. (2013)^c,d^	Detry et al. (2015)	Kim et al. (2016)	Lee et al. (2016^)c^	Ye et al. (2017)	Kang et al. (2019)^a,c^
Patients with recurrence (events)	23	28	5	30	n.s	53	74
Number of variables in multivariate COX analysis	3	11	3	5	13	9	4
Events per independent variable	7.7	2.5	1.6	6	n.s	5.9	18.5
Milan	–	n.s	–	0.029	–	0.004	–
AFP	–	n.s	–	–	0.991	0.001	< 0.001
Number of lesions	–	n.s	–	–	0.534	0.485	0.046
Size of lesions	–	n.s	n.s	–	0.001	–	0.003
Grade	0.044	n.s	n.s	–	0.927	0.380	–
pV	–	n.s	–	–	0.033	< 0.001	–
pT-category	< 0.001	–	–	–	–	–	–
Tumor to liver SUV ratio	0.031	0.024	0.018	0.009	0.001	0.011	< 0.001

– Not included in COX analysis, *n.s.* not stated

^a^Multicentric

^b^Mean follow-up

^c^Only living donor liver transplantation

^d^3 year rates presented

Five of the six studies listed in Table 4 come from Asia (Kang et al. 2019; Kim et al. 2016; Lee et al. 2013, 2016; Ye et al. 2017), three of the studies have mean or median follow-up intervals < 30 months, three studies present results after living donor liver transplantation. The influence of different PET/CT scanners on the results is unclear. Before this background, it seems to early to perform a metaanalysis with combined statistics.

Therefore, there is an urgent need for studies with larger sample sizes and standardized documentation to overcome the methodical problem of small numbers of recurrences in limited sample sizes.
